# Hair cortisol analyses in different mammal species: choosing the wrong assay may lead to erroneous results

**DOI:** 10.1093/conphys/coaa009

**Published:** 2020-02-28

**Authors:** Katarina Jewgenow, Alexandre Azevedo, Mareen Albrecht, Clemens Kirschbaum, Martin Dehnhard

**Affiliations:** 1 Department Reproduction Biology, Leibniz Institute for Zoo and Wildlife Research, Alfred-Kowalke-Str.17, D-10315 Berlin, Germany; 2 Instituto de Ciências Biomédicas Abel Salazar, R. Jorge Viterbo Ferreira 228, 4050-313 Porto, Portugal; 3 Faculty of Psychology, Department of Biopsychology, Technical University of Dresden, Helmholtzstraße 10, D-01069 Dresden, Germany Germany

**Keywords:** glucocorticoids, hair, HPLC, immunogram, LC-MS/MS

## Abstract

Wild animals are faced with a broad range of environmental stressors and research is needed to better understand their effect on populations. Hormone analysis based on enzyme immunoassays (EIAs) can provide valuable information on adrenocortical activity (stress), and assessment of cortisol in hair may allow the quantification of cortisol production. To validate hair hormone analysis, we compared two EIAs based on antibodies against cortisol-3-CMO-BSA and cortisol-21-HS-BSA for hair glucocorticoid (hGC) measurements in Egyptian mongoose, Iberian lynx, Alpine marmot, Asiatic black bear, spotted hyena and cheetah, with results obtained by liquid chromatography coupled with tandem mass spectrometry (LC-MS/MS) measurements. Both EIAs were also characterized by HPLC immunograms. Our results revealed that the cortisol-21-HS EIA measured 2.3- to 12-fold higher hGC concentrations than the cortisol-3-CMO assay. In dependence of the species, high-performance liquid chromatography (HPLC) immunograms showed that up to 70% of immunoreactivities determined by the cortisol-21-HS constituted of unknown unpolar compounds leading to an overestimation of hGC. The cortisol-3-CMO EIA expressed a better specificity, with 32.1–67.4% of immunoreactivity represented by cortisol and cortisone. The LC-MS/MS analyses (gold standard) revealed that the cortisol-3-CMO EIA also resulted in an (up to 3-fold) overestimation of hGC, but EIA results were correlated with LC-MS/MS in the mongoose, the lynx, the spotted hyena and the marmot. No correlation was obtained for Asiatic black bears. As a result of our study, we strongly recommend to test any cortisol EIA for its specificity towards extracted hair components. In all analyzed species, except the Asiatic black bear, cortisone and cortisol were simultaneously present in hair extracts; consequently, an appropriate EIA should cross-react to these two glucocorticoid hormones and express negligible affinity towards substances with less polarity than corticosterone. Choosing the wrong EIA for hGC analyses may lead to overestimations of hGC or—in the worst case—to results that do not mirror real adrenocortical activity.

## Teaser

We analyzed glucocorticoids from hair of six different mammalian species and found that choosing the wrong assay may lead to overestimations in comparison with the steroid amount determined by mass spectrometry. With hair glucocorticoids being used as a marker of stress this can, in the worst case, lead to erroneous conclusions

## Introduction

Wild animals are faced with a broad range of environmental stressors, and one physiological process helping vertebrates to cope with any challenge is the stimulation of the hypothalamic pituitary axis (HPA) leading to the release of glucocorticoid (GC; corticosterone/cortisol) hormones. These metabolic hormones not only are involved in the maintenance of energetic balance but also act as key components of the acute stress response. Baseline levels of GC in free-living animals may vary during development and throughout the year. Thus, annually varying GC levels reflect the physiological response of animals to meet seasonal needs, which are thought to be different between sexes (Romero, 2002; Bauer *et al.*, 2014).

In case of acute stress events, however, increased levels of GC are essential to shift energy towards the functions demanded to rapidly cope with the stressor (amongst others dominance behaviour, intra- and inter-species competition, escape from predators and human–wildlife conflicts) (McEwen *et al.*, 2003). Usually, these changes are of limited duration, and thereafter GC levels return to baseline, but if adverse environmental conditions act permanently, continuously elevated GC secretion may cause deleterious consequences by altering individual fitness, immune function and reproductive success (Terio *et al.*, 1999; Tilbrook *et al.*, 1999; Ludwig *et al.*, 2019) and eventually reduce survival (Wingfield *et al.*, 2003). Thus, the possible impact of an environmental stressor, either of anthropogenic or of natural origin on an animal’s fitness, can only be detected if physiological baselines are known and reliable measurements of GC are performed on a regular and large-scale basis.

Determination of cortisol as an indicator of the hypothalamic–pituitary–adrenal (adrenocortical) activity is often used to measure stress. Since serum GC concentrations are characterized by diurnal pulsatile fluctuations and capture/restraint for blood collection induce increases of GC concentration within a few minutes (Carnes *et al.*, 1988; Sheriff *et al.*, 2011), alternative approaches that use matrices like urine, faeces or hairs have been developed and validated for wild animals.

Urine and faeces can be collected non-invasively from terrestrial mammals without causing stress from capture, restraint and venipuncture procedures, mirroring adrenocortical (stress) status of the preceding hours or days. Urine, however, requires special sampling procedures in captive animals and is almost impossible in free-ranging animals. Faecal samples are easy to obtain from wild animals and DNA-based technologies can be applied (Sheriff *et al*., 2011) to assign them to a species or even to individuals (Mohd Salleh *et al.*, 2017; Mengulluoglu *et al.*, 2019). In particular, faecal glucocorticoid metabolites (fGMs) are described as reliable indicators of GC excreted by the adrenal glands during the previous 12–24 h (depending on defecation rates). For some species, the delay may be even longer (e.g. reptiles). They are less affected by episodic fluctuations or the pulsatility of hormone secretion and are therefore useful to evaluate adrenal activity in an integrated manner (Sheriff *et al.*, 2011; Palme, 2019). However, successful measurement of fGM relies on the development of immunoassays that detect the species-specific hormone metabolites of cortisol or corticosterone. The native hormones are, if at all, represented in minor quantities in faecal samples, as has been shown, e.g. for carnivores (Young *et al.*, 2004). In most cases, fGM still cross-react with antibodies primarily produced to measure the respective unmetabolized GC in the plasma. However, experiments dealing with the physiological and biological validity of fGM analyses are essential to prove whether an immunoassay is able to reflect an animal’s endocrine status. The most widely used approach is to stimulate adrenocortical activity with an adrenocorticotropic hormone (ACTH) challenge test or to measure an increase of fGM in response to a known stressor experienced by the animal (Touma *et al.*, 2004; Young *et al.,* 2004).

In contrast to fGM, assessment of cortisol in hair may reflect circulating cortisol levels over extended time periods, although the mechanism of incorporation is still a matter of debate. It is suggested that cortisol is incorporated into hairs by passive diffusion from the bloodstream and therefore may serve as a unique biomarker of HPA activity providing an integrated value of circulating hormones covering several weeks or even months (Davenport *et al.*, 2006; Accorsi *et al.*, 2008; Macbeth *et al.*, 2010; Ashley *et al.*, 2011; Gonzalez-de-la-Vara *et al.*, 2011; Terwissen *et al.*, 2013; Mastromonaco *et al.*, 2014). Consequently, hair cortisol is thought to be insensitive to the impact of sampling procedures, ultradian rhythms and the effect of any acute stressor, which certainly offers great potential as an indicator of chronic stress. However, a recent radio-metabolism study using labelled cortisol demands a cautious interpretation of the retrospective time frame to record stress in relation to the hair growth rate (Kapoor *et al.*, 2018). The authors also showed that radiolabeled cortisol was incorporated into hair of rhesus monkey as cortisol and cortisone and as other unknown metabolites, whereas in the guinea pig radiolabeled cortisol was incorporated mainly as cortisone (Keckeis *et al.*, 2012). Raul et *al.* (2004) suggested for human that the enzyme 11β-hydroxysteroid-dehydrogenase (HSD) (in)activates GCs converting cortisol to cortisone, and a conversion of cortisol to cortisone before its incorporation in hair (Raul *et al.*, 2004). Thus, in addition to cortisol, cortisone may also be regarded as a useful biomarker for stress research.

The authentic hormones should be detectable when applying an immunoassay. However, the application of antibody-based methods requires a thoughtful validation before they can provide useful information on the effect of stressors on animals. For example, the body regions from which samples are collected may influence the concentration of cortisol present in hair (Terwissen *et al.,* 2013; Acker *et al.*, 2018). Mechanical irritation by scratching might enhance local hair cortisol production as well (Salaberger *et al.*, 2016). Moreover, hair growth pattern might be highly variable between individuals, which may affect between subject comparisons (Kapoor *et al.,* 2018). In addition, the precise mechanisms by which lipophilic steroid hormones are incorporated into hair are still not fully understood. *In vitro*, an equivalent of the central HPA in the hair follicle of humans has been demonstrated (Ito *et al.*, 2005). Data from a radiometabolism study performed in guinea pigs (Keckeis *et al.*, 2012) demonstrated only little amounts of injected 3H-cortisol in the hair matrix, but surprisingly high amounts of unlabeled cortisol, assuming a local synthesis and quite likely an autonomous production of cortisol (Cirillo *et al.*, 2011). In addition, reversed-phase high-performance liquid chromatography (HPLC) of hair samples from guinea pig and wool samples from sheep demonstrated-depending on the applied GC enzyme immunoassay (EIA)-the presence of cortisol, cortisone and corticosterone as well as of several unknown cortisol immunoreactivities (Keckeis *et al.*, 2012, Salaberger *et al.,* 2016).

These unknown steroid hormones may hamper the determination of GC in hair samples (hGC) leading to an overestimation of GC content in hair samples due to unforeseeable cross-reactivities with the antibody. The application of high-performance liquid chromatography coupled with tandem mass spectrometry (LC-MS/MS) for exact quantification of hGC is therefore preferable to any immune assay. It combines the physical separation of liquid chromatography (LC) paired with the specificity of mass analysis capabilities of tandem mass spectrometry (MS/MS) allowing the sensitive detection and quantification of multiple compounds. Until now, only a few LC–MS/MS methods for the simultaneous identification and quantitation of steroids in hair have been described (Stalder *et al.,* 2013). In contrast to an EIA, LC-MS/MS equipment is quite expensive and demands high analytical skills. Therefore, it is often unavailable to smaller laboratories and, in particular, to researchers working on wildlife. For them, immunoassays are still the method of choice.

We recently validated an EIA to determine hair cortisol concentrations in wild caught Egyptian mongoose (Azevedo *et al.*, 2019). In the present follow-up study, we applied two different cortisol EIA, and obtained different cortisol concentrations in identical hair extracts in the mongoose and five other wildlife species. By conducting HPLC immunograms, we determined the immunoreactive components in hairs and related them to known steroids, in particular to GC. This allowed us to identify the predominant hGC component in the six species. Finally, we assessed the steroid composition by LC-MS/MS analyses (gold standard) and analyzed the EIA quantification in light of the LC-MS/MS results.

## Materials and methods

All chemical reagents were purchased from Sigma-Aldrich (Taufkirchen, Germany) unless stated otherwise and were of the highest purity available.

### Sample collection

Hair samples were obtained from six mammalian species in the framework of different research programs: Egyptian mongoose (*Herpestes ichneumon*), Iberian lynx (*Lynx pardinus*), cheetah (*Acinonyx jubatus*), Alpine marmot (*Marmota marmota*), Asiatic black bear (*Ursus thibetanus*) and the spotted hyena (*Crocuta crocuta*).

Hair samples from the individual Egyptian mongoose (*Herpestes ichneumon*; *n* = 294; 140 males and 154 females; 147 adults and 97 juveniles and sub-adults) were collected from hunting activities (Bandeira *et al*., 2016) and were previously used to determine baseline variation in hair cortisol levels (Azevedo *et al*., 2019). Following death, specimens were frozen at −20°C until the date of sample collection. After thawing, hair was clipped with scissors from a standard area between the shoulders and stored in paper envelopes until further processing (Azevedo *et al.,* 2019). Samples from individual Iberian lynx (*Lynx pardinus*; 93 adult females, 6 adult males) were obtained during routine health checks within the Iberian lynx conservation breeding program in Spain and Portugal. The hair was clipped from the inner surface of hind legs.

All other hair samples were obtained from the IZW hair collection compiled over several years. Hair samples from 20 alpine marmot (*M. marmota*) were clipped from an area between shoulders (5 adult males, 4 adult female, 11 juveniles) housed at the Feldstation Niederfinow 70 km north of Berlin. The samples from adult spotted hyenas (*C. crocuta; n* = 7; 2 males, 5 females) and from the one adult male cheetah were collected from the tail region, whereas those from adult Asiatic black bears (*U. thibetanus*; *n* = 14; 9 males, 5 females) were obtained from the inner surface of hind legs. All hair samples were stored in envelopes at room temperature.

From all Iberian lynx and Egyptian mongoose hair samples, an aliquot of 20 mg was separated and washed twice with 2 ml of 90% methanol by vortexing for 5–10 s to remove surface contaminations. Thereafter, the samples were dried for 1 h at 70°C and aliquots (~10 mg) from each Egyptian mongoose (*n* = 294) and female Iberian lynx (*n* = 93) were taken and milled to a fine power with ceramic beads in a tissue homogenizer as described before (Azevedo *et al*., 2019). Then, 400 μl of 90% methanol was added to the powder and shaken at room temperature for 30 min. Following centrifugation (3 min, 1000×g), the supernatant was collected and transferred into a new tube, diluted 1:2 with water and kept frozen until EIA analysis and the preparation of HPLC immunograms. From hair samples of 14 mongooses (7 males, 7 females) and 12 lynxes (6 males, 6 females), additional extracts were produced for LC-MS/MS analyses (see below) following the same protocol.

For hair samples from the Asiatic black bear, the alpine marmot, the cheetah and the spotted hyena, the washing procedure was modified utilizing aliquots of 50–60 mg that were washed twice with 4.5 ml of 90% methanol. Thereafter, the samples were extracted as described by Carlitz *et al*. 2014. In brief, before washing and drying, hairs were cut into 10-mm pieces and afterwards 50 mg of the dried hairs were incubated with 1.8 ml of methanol for 18 h at 45°C. Subsequently, the extract was aspirated, dried for 20 min at 60°C under a constant stream of nitrogen and resolved in 1 ml 90% methanol. An aliquot of 100 μl was diluted 1:2 with water for EIA and kept frozen. The remaining 900 μl was subjected to LC-MS/MS analyses or were pooled for HPLC-immunograms (see below).

### GC analyses

We used two in-house immunoassays based on polyclonal antibodies (rabbit) directed against (i) cortisol-3-CMO-BSA and (ii) cortisol-21-hemisuccinate (HS)-BSA. The corresponding label of cortisol were cortisol-3-CMO-peroxidase and cortisol-21-HS-peroxidase as previously described in (Ludwig *et al.*, 2013) and in (Kelm *et al.*, 2016), respectively. The antibody cross-reactivities of the cortisol-3-CMO assay were as follows: cortisol, 100%; cortisone, 19.5%; corticosterone, 6.3%; desoxycorticosterone, 0.1%; progesterone, 0.1%; estradiol, 0.1%; testosterone, 0.1%. Those of the cortisol-21-HS EIA were cortisol 100%; cortisone, 17.5%; progesterone, 13.2%; corticosterone, 10.4%; desoxycorticosterone, 7.9%; estradiol, 0.1%. The principle of our in-house EIA procedure has been described in detail in Finkenwirth *et al.* (2010). All EIA measurements were conducted in 20 μl duplicates and results expressed as pg/mg hair weight.

For both EIA applied, serial dilutions of hair extracts showed parallelism to the cortisol standard with no significant difference in slopes (*P* > 0.05). The sensitivity of the assays was 0.40 pg/well. The inter-assay and intra-assay coefficients of variation were determined on two pooled extracts. For cortisol-3-CMO EIA, the pools contained 0.25 and 0.6 ng/ml cortisol. Inter-assay and intra-assay coefficients of variation were 11.3% (*n* = 7) and 9.5% (*n* = 7), and 6.7% (*n* = 16) and 5.4% (*n* = 16), respectively. For the cortisol-21-HS assay, the inter-assay and intra-assay coefficients of variation were determined on two different extraction pools, which were composed of 0.25 ng/ml and 0.5 ng/ml cortisol. The corresponding inter- and intra-assay variations were 14.6% (*n* = 7) and 9.8% (*n* = 8), and 12.8% (*n* = 16) and 5.9% (*n* = 16), respectively.

### High-performance liquid chromatography

To characterize the compounds detected by the respective EIA, we ran HPLC immunograms from pooled hair extracts that were obtained using two different extraction procedures (see above). From the hair extracts of 294 mongooses and 93 female lynxes, aliquots of 150 and 460 μl of each extract were pooled corresponding to ~550 and 530 mg of hairs, respectively. For the marmots (*n* = 20) and black bears (*n* = 14), 50 mg hair of each individual was extracted as described and 900-μl aliquots of each extract were combined, resulting in a pool of ~900 and 630 mg of hairs, respectively. From each individual hyena samples (*n* = 7), two different hair aliquots of 50 mg were extracted, whereas from the one cheetah 20 hair aliquots of 50 mg were taken. From each extraction, 900 μl was pooled, so the pools for hyena and cheetah represented ~630 and 700 mg of hairs, respectively. All pools were purified on Octadecyl C18 columns (0.5 ml, J.T. Baker, BAKERBOND SPE™ 7020-01) as described in Dehnhard *et al.* (2010) and Azevedo *et al.* (2019). Eluates were evaporated in a heater at 55°C under nitrogen and reconstituted in 200 μl of 40% methanol. One hundred to 150 μl portions of purified hair extract were used for HPLC analysis on a reverse-phase Ultrasep ES100/RP-18/6 μm HPLC column (4 × 250 mm, Sepserv, Berlin). Compounds were separated using a methanol + water mixture with the following gradient: 60% methanol over 5 min, 60–90% methanol over 10 min and 90–100% methanol over another 10 min. The flow rate was 1 ml/min. Fractions of 0.33 ml were collected at 20-s intervals over a period of 25 min. All fractions were lyophilized and re-suspended in 200 μl 40% methanol before 20-μl aliquots were determined by both cortisol-EIA. The elution positions of native cortisone, cortisol, corticosterone, 11-hydroxyetiocholanolone, testosterone (T), epi-androsterone, dihydrotestosterone (DHT) and progesterone (P4) on this column had been previously determined in separate HPLC runs (Ludwig *et al.,* 2013; Pribbenow *et al.*, 2015).

### Liquid chromatography coupled with tandem mass spectrometry

Hair extracts from individual mongooses (*n* = 14), Iberian lynxes (*n* = 12), marmots (*n* = 20), Asiatic black bears (*n* = 14) and spotted hyenas (*n* = 7) were adjusted to an absolute content about 150 pg cortisol. To achieve this, cortisol concentrations of extracts were determined by 3-CMO EIA, and the required volume of each species was determined according to the mean cortisol concentration (Table 2). Thus, for mongoose 300 μl (~7.5 mg hair), for Iberian lynx 200 μl (~5 mg hair), for marmots 600 μl (~30 mg hair) and for black bears 800 μl (~40 mg hair) of each extract were transferred to 1.5-ml reaction tubes, dried down for 90 min at 60°C under a constant stream of nitrogen and dissolved in 225 μl 50% methanol (LC-MS/MS grade). The samples were sent to the Department of Biopsychology, Technical University of Dresden, Germany, for LC-MS/MS analyses. Details on liquid chromatography methodology and mass spectrometric conditions are described in Carlitz *et al.* (2016) and Gao *et al*. (2013).

### Statistical methods

Determination of mean values ± SD, comparison of cortisol amounts between both EIA as well regression analysis were performed using IBM SPSS Statistics 24 (SPSS Inc., IBM, Armonk, USA). Significance was set to *P* < 0.05. Tukey box plots were produced by SigmaPlot for Windows (Version 10.0).

Paired *t* test for comparison of means based on two-tailed *P* values was applied after testing for normality. Pearson correlation coefficients were calculated to detect the relation between the two cortisol EIA (Supplement Table 1). Regression analysis was also performed between the hGC amount determined by LC-MS/MS (sum of cortisol and cortisone) in relation to the amount determined by EIA (Supplement Table 2).

**Table 1:** hGC concentrations in five different mammalian species determined by two different cortisol EIA, a cortisol-3-CMO and a cortisol-21-HS. Represented are means ± SD. For correlation analysis, Pearson’s correlation coefficients (*r*) were determined

Species (Number)	Cortisol-21-HS in pg/mg Mean ± SD (Range)	Cortisol-3-CMO in pg/mg Mean ± SD (Range)	Relation	correlation coefficient *P* value
Egyptian mongoose (294)	159.7 ± 46.4 (61.9–394.6)	20.0 + 8.5 (0.0–114.2)	8: 1	*r* = 0.324 ***P* = 0.000**
Iberian lynx (12)	87.7 ± 46.9 (32.2–174.3)	37.3 ± 13.2 (21.1–60.5)	2.3: 1	*r* = 0.863 ***P* = 0.000**
Alpine marmot (20)	60.6 + 18.6 (36.3–111.5)	6.0 + 1.9 (4.1–11.5)	10: 1	*r* = 0.190 *P* = 0.423
Asiatic black bear (14)	44.6 + 20.2 (15.5–76.2)	3.7 ± 2.3 (0.8–10.6)	12: 1	*r* = 0.619 ***P* = 0.009**
Spotted hyena (7)	28.9 + 16.8 (18.0–60.7)	12.1 ± 2.7 (6.6–17.5)	2.3: 1	*r* = 0.507 *P* = 0.246

Differences in mean for cortisol levels determined by cortisol-21-HS and cortisol-3-CMO EIA were highly significant (*P* < 0.001) for the Egyptian mongoose, the Iberian lynx, the marmot and the Asiatic black bear, whereas in case of the spotted hyena the difference was significant (*P* = 0.036). The respective *t* values (degree of freedom) in paired *T* test were 56.039 (293), −4.833 (11), −13.400 (17), 7.830 (13) and −2.684 (6) for the mongoose, lynx, marmot, black bear and hyena, respectively.

As described before in Azevedo *et al*. (2019) for the results of cortisol-3-CMO EIA, the statistical analysis of the effects of age, season, sex and storage on hair cortisol measured with cortisol-21-HS EIA was performed in R (v3.5.1) using linear mixed effects models with a Gaussian error distribution using the package lme4 (Bates *et al*. 2015), and variable significance (*α* = 0.05) was determined with parametric bootstrapped likelihood ratio tests with 5000 iterations using the package pbkrtest (Halekoh and Højsgaard, 2014).

Linear mixed effects models and likelihood ratio tests were performed on data without outliers for cortisol-3-CMO EIA (taken from Azevedo *et al*. 2019) and 21-HS-cortisol EIA. Values four times larger than median hair cortisol and almost twice as high as the next highest measurement were defined as outlier. Four-fold increases in hair cortisol have previously been observed in response to repeated ACTH challenge in dairy cattle (Gonzalez-de-la-Vara *et al*. 2011) and eastern chipmunks (Mastromonaco *et al*. 2014), and so these values may be biologically plausible in situations of chronic and severe stress.

## Results

### Determination of cortisol content in hair samples using EIA

**Figure 1:**
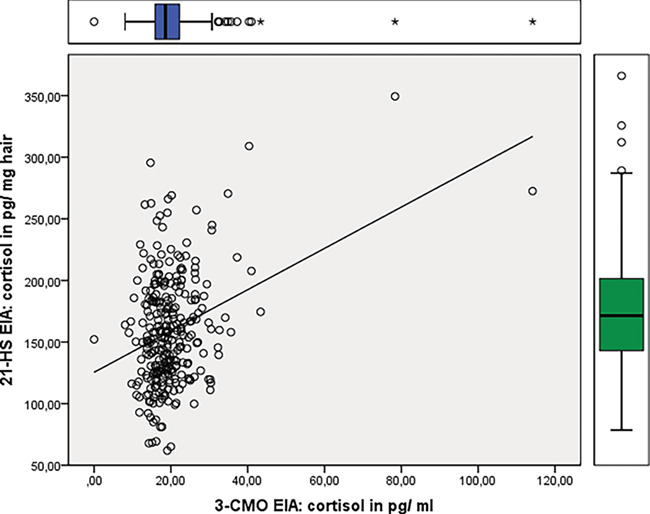
X-Y-diagram of cortisol data determined with 3-CMO EIA versus 11-HS EIA for all samples from mongoose, combined with box-plots of both EIA.

Within a framework of other projects, 294 hair samples from Egyptian mongoose were analyzed with both cortisol EIA revealing significant differences between both EIA (Table 1, Supplement Table 1, paired *t* test: *t* = 56.039, df = 293, *P* < 0.0001). The mean cortisol content determined with cortisol-21-HS EIA was about eight times higher compared to the results obtained with the cortisol-3-CMO assay; both data sets were correlated with each other (Table 1, Fig. 1, *r* = 0.324, *P* < 0.0001). By applying linear mixed fixed models for data obtained with cortisol-3-CMO EIA it was shown that age, sex and storage time had an effect on hair cortisol, but season did not. The full data analysis was reported in Azevedo *et al*. (2019). In contrast, the same analysis performed on hGC concentrations determined with cortisol-HS-21 EIA revealed that only age had an effect (Table 2, Supplement Table 3, Supplement Fig. 1).

**Table 2:** Significance of model terms age, season, sex and storage time, calculated according to Azevedo *et al*. (2019) using parametric bootstrapped likelihood ratio tests, with results of cortisol-21-HS and cortisol-3-CMO EIAs, respectively

**Cortisol-21-HS**			**Cortisol-3-CMO**	
**Variable**	**Likelihood ratio test value**	***P* value**	**Likelihood ratio test value**	***P* value**
Age	**3.052**	**0.029**	19.38	**0.001**
Season	2.039	0.109	5.71	0.187
Sex	0.086	0.769	**5.05**	**0.028**
Storage time	0.180	0.671	**9.51**	**0.020**

Likelihood ratio test value is the value of the likelihood ratio test value generated from the true data, which was then compared to likelihood ratio test values simulated with parametric bootstrapping. Significant terms (*α* = 0.05) are in bold.

In the Iberian lynx and the spotted hyena (Table 1, Supplement Table 2), the difference between the two cortisol EIA was less pronounced with less than half cortisol determined when using the cortisol-3-CMO assay (lynx: *t* = −4.833, df = 11, *P* < 0.0001; hyena: *t* = −2.684, df = 6, *P* = 0.036). From the samples from marmots and Asiatic black bears (Table 1, Supplement Table 2), we also obtained significantly different hGC measurements between the two EIA. Analyses with the cortisol-21-HS EIA revealed 10- and 12-fold higher hGC concentrations in the marmot (*t* = −13.400, df = 19, *P* < 0.0001) and the black bear (*t* = 7.830, df = 13 *P* < 0.0001), respectively. Although being significantly different between the EIAs, results of both EIA were still correlated in the Egyptian mongoose, the Iberian lynx and the Asiatic black bear, whereas no relation between both EIA was found in alpine marmots and spotted hyenas (Table 1).

### LC-MS/MS analyses

LC-MS/MS allows the selective detection of different GCs in hairs combining the physical separation of liquid chromatography (HPLC) paired with the specificity of tandem mass spectrometry (MS/MS) and was therefore used to assess the results obtained by the two different EIA. In our study, quantitative LC-MS/MS determinations of cortisone, cortisol and corticosterone were carried out in a total of 68 hair samples from the above mentioned species (limit of detection [LOD] = 0.32 pg/mg, limit of quantification [LOQ] = 1.05 pg/mg). In all samples, the cortisol content was determined by both EIA as well (Table 3, Supplement Table 2) making species differences apparent.

Highest cortisone and cortisol concentrations were found in the lynx followed by lower concentrations in the mongoose and the hyena. Mean cortisol concentrations below 1.0 pg/mg were obtained in the marmot and the black bear. Moreover, cortisol levels in 9, 3 and 10 samples were below detection limit in the mongoose, the marmot and the black bear, respectively. Except from the black bear, low corticosterone concentrations were obtained in all species and were completely undetectable in the lynx and the marmot. Although almost close to detection level, corticosterone was measurable in all black bear samples. Whether corticosterone represents the main hGC in the black bear needs to be confirmed.

**Table 3:** hGC concentrations achieved by comparative LC-MS/MS determination of cortisone, cortisol and corticosterone in a total of 68 hair samples from six mammal species. In addition, cortisol concentrations were determined by both EIA, cortisol-3-CMO and cortisol-21-HS, respectively. Represented are means ± SD. For correlation analysis, Pearson’s correlation coefficients (*r*) were determined between the sum of cortisol+cortisone and results of both EIA, respectively

	LC-MS/MS				hGC by EIA		Correlation coefficient; *P*-value			
Species(Number)	Cortisone(pg/mg)	Cortisol(pg/mg)	Corticosterone(pg/mg)	Cortisone+ cortisol(pg/mg)	21-HS EIA(pg/mg)	3-CMO EIA(pg/mg)	21-HS EIA vs. cortisol+cortisone		3-CMO EIA vs. cortisol+cortisone	
Egyptian mongoose (14)	6.11 ± 3.66	5.31 ± 9.39	1.81 ± 1.95	11.43 ± 12.63	193 ± 68.6	20.6 ± 15.0	r = 0.691	***P* = 0.000**	r = 0.914	***P* = 0.000**
Iberian lynx (12)	24.82 ± 9.58	13.35 ± 7.80	0.00	38.16 ± 12.27	87.8 ± 46.9	37.3 ± 13.2	r = 0.799	***P =* 0.001**	r = 0.855	***P* = 0.000**
Spotted hyena (7)	3.03 ± 1.21	4.41 ± 1.33	0.36 ± 0.62	7.44 ± 2.24	28.9 ± 16.8	13.6 ± 4.1	r = 0.5	*P =* 0.127	r = 0.807	***P* = 0.014**
Alpine marmot (20)	0.90 ± 0.65	1.14 ± 1.47	0.00	2.03 ± 2.1	60.6 ± 18.6	6.04 ± 1.92	r = −0.087	*P* = 0.358	r = 0.847	***P* = 0.000**
Asiatic black bear (14)	0.36 ± 0.13	0.06 ± 0.10	2.02 ± 1.18	0.42 ± 0.13	50.8 ± 22.6	3.7 ± 2.4	r = −0.252	*P* = 0.193	r = 0.10	*P =* 0.367
Cheetah (1)	7.2	3.7	1.2	10.9	55.5	13.8	-	-	-	-

### Comparison between cortisol EIA and LC-MS/MS analyses

**Figure 2:**
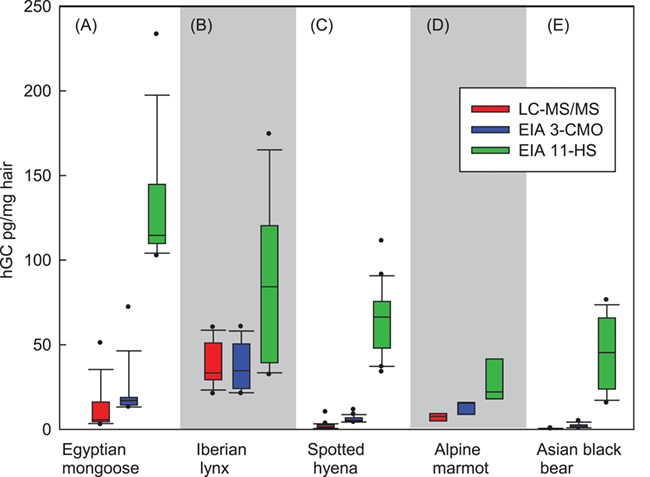
Box plots of hair cortisol content determined by LC-MS/MS (sum of cortisol and cortisone) and by two cortisol EIA, cortisol-3-CMO EIA and cortisol-21-HS EIA.

Altogether, LC-MS/MS analyses revealed similar species differences compared to the analyses with both EIA (Table 3, Fig. 2). In the lynx, the highest amounts of hGC were detected, and the range of LC-MS/MS concentrations expressed as the sum of cortisone + cortisol (38.16 ± 12.27 pg/mg) matched with the mean values obtained by the 3-CMO EIA (37.3 ± 13.2 pg/mg, Table 3).

In hair samples of mongoose (Table 3), the LC-MS/MS cortisone + cortisol content was determined to be 11.43 ± 12.63 pg/mg. In comparison, the 3-CMO EIA overestimated hGC twice (20.6 ± 15.0 pg/mg), whereas the cortisol-21-HS EIA revealed an about 10-fold higher overestimation (193.15 ± 68.65 pg/mg) of the same sample set. Similar results were obtained for the marmots, where 21-HS EIA concentrations (60.6 ± 18.6 pg/mg, Table 3) did not reflect the hGC determined by LC-MS/MS at all, whereas the use of the 3-CMO EIA revealed three times higher values (2.03 ± 2.1 pg/mg LC-MS/MS vs 6.04 ± 1.92 pg/mg 3-CMO, Table 3). In the hyena, the mean 3-CMO EIA amount (13.58 ± 4.12 pg/mg) was just double, whereas the 21-HS EIA amount was 4-fold higher than the amount determined by LC-MS/MS (7.44 ± 2.24 pg/mg). In the black bear samples, almost no cortisone and cortisol were detectable by LC-MS/MS. This coincides with values obtained by the 3-CMO EIA that were the lowest for all examined species (Table 3), whereas the results obtained by 21-HS EIA were more than 10 times higher (50.8 ± 22.6 pg/mg) and thus were in the same range as those determined for lynx, hyenas and marmots with this assay (Fig. 2, Table 3). 

With the limited samples available, we also performed a regression analysis between results obtained by 3-CMO EIA, 21-HS EIA and the LC-MS/MS concentration of the sum of cortisone + cortisol (Table 3). The results indicated for a significant relationship between LC-MS/MS and 3-CMO analysis in case of the mongoose, the lynx, the hyena and the marmot (Table 3), but only for the mongoose and the lynx a significant relation was found between LC-MS/MS and 21-HS EIA results. For the black bear, both EIA results were not correlated to the sum of cortisol + cortisone analyzed by LC-MS/MS. This might be related to the very low hGC concentration determined by LC-MS/MS.

### HPLC analyses of immunoreactive GC in hair samples

**Figure 3:**
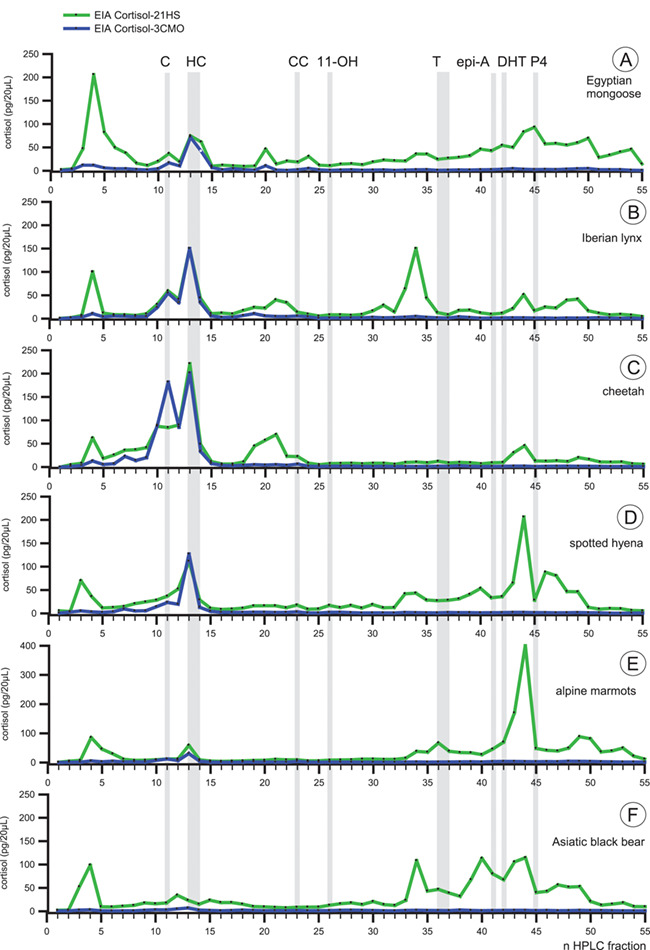
High-performance liquid chromatography (HPLC; reversed phase) separations of immunoreactive cortisol metabolites in pooled hair samples from six different mammalian species. The obtained fractions were analyzed with a cortisol-3-CMO EIA (blue lines) and a cortisol-21-HS EIA (green lines). The elution positions of reference standards are indicated by vertical lines; 11: C (cortisone); 13/14: HC (cortisol); 23: CC (corticosterone); 26: 11-OH (11-hydroxyetiocholanolone); 36/37: T (testosterone); 41: epi-A (epi-androsterone); 42: DHT (dihydrotestosterone); 45: P4 (progesterone). Pooled samples of A: Egyptian mongoose (*Herpestes ichneumon*, *n* = 294, 550 mg hair), B: Iberian lynx *(Lynx pardinus*, *n* = 93, 530 mg hair), C: cheetah (*Acinonyx jubatus*, *n* = 1, 900 mg hairs), D: spotted hyena (*Crocuta crocuta*, *n* = 7, 630 mg hair), E: marmot (*Marmota marmot*, *n* = 20; 900 mg hair) and F: Asiatic black bear (*Ursus thibetanus*, *n* = 14, 630 mg hair) were used.

To further elucidate the differences between both cortisol-EIA, we performed HPLC immunograms to characterize the immunoreactive compounds in hair extracts of six mammalian species (Fig. 3). For the six species analyzed, very different results were obtained, reflecting the specificity of both cortisol antibodies towards different compounds extracted from mammalian hairs. In all samples, the 3-CMO EIA detects immunoreactive substances in Fraction 11 and, more pronounced, in Fraction 13–14, which coincide with elution peaks of cortisone and cortisol, respectively. The cortisol-21-HS antibody, however, has been found to be less specific, since it additionally cross-reacted with unknown compounds in Fractions 3–7, and depending on species, with substantial amounts of unpolar substances in fractions above 33.

**Figure 4:**
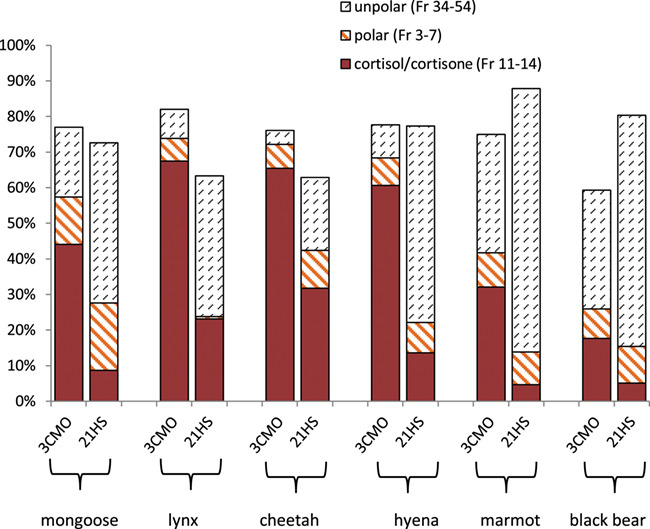
Comparison of HPLC immunograms from six mammal species showing the percentage of compounds contributing to the HPLC-immunograms. Fractions 3–7: polar, probably conjugated GCs; Fractions 11–14 cortisol and cortisone; Fractions 34–54: unpolar compounds.

In detail, in mongoose hair extracts (Fig. 3A) the proportions of cortisone and cortisol together represent only 10.5% of the total immunoreactivity when using the cortisol-21-HS EIA. Despite a major peak between Fractions 3–7, most immunoreactivity was found contributing to an unclear profile with peaks in Fractions 45, 50 and 54 not corresponding to any of our steroid standards. Moreover, some of them were less polar than our most unpolar standard in Fraction 45 (progesterone). Clearly different results were achieved with the cortisol-3-CMO assay, identifying cortisol (Fraction 13/14; 34.6%) and cortisone (Fraction 11; 10.9%) as the most prominent peaks. In addition, a polar peak in Fractions 3–6 has been found (11.8%), probably representing conjugated GCs. Following two minor peaks in Fractions 20 and 24, the profile nearly runs on baseline without the presence of questionable unpolar compounds. No significant peaks were detected on the position of corticosterone (Fraction 23). HPLC-immunograms from the lynx (Fig. 3B) clearly revealed two major peaks in Fractions 11 and 13 representing the elution positions of cortisone and cortisol, respectively, when using the cortisol-3-CMO assay, escorted by marginal amounts of additional compounds. For comparison, the cortisol-21-HS assay detects similar quantities of cortisone and cortisol; however, substantial amounts of additional less polar immunoreactivities were recognized in Fractions 18–22, 31, 33–35 and between 43–50. None of these immunoreactivities fits with the elution position of any of our standards. Altogether, the cortisol-3-CMO EIA specifically identifies cortisone and cortisol, both representing 67.4% of total immunoreactivity.

Similar deviating immunograms were found when checking both EIA in samples from the cheetah (Fig. 3C). As in the lynx the cortisol-21-HS EIA depicts considerable amounts of unknown compounds, whereas the use of the corresponding 3-CMO EIA increases the specificity towards cortisone + cortisol up to 65.4%.

A similar difference between both cortisol-EIA was found for hair samples from the spotted hyena (Fig. 3D). Again, the cortisol-21-HS assay cross-reacted with unpolar compounds eluting between Fractions 33 and 49 and peaking in Fraction 44 close to the elution position of progesterone. The cortisol-3-CMO assay only depicts cortisone and cortisol which together make up 60.6% of the total immunoreactivity.

In the marmot, the differences between both EIA were even more profound (Fig. 2E). From the total amount of immunoreactivity detected by the 21-HS assay, only a very small proportion (3.5%) was comprised of cortisol (Fraction 13) and probably cortisol conjugates (Fraction 4–6). Moreover, the 21-HS assay detected unpolar immunoreactivities with a peak in Fraction 44. Again, the 3-CMO assay was found to be more specific detecting cortisol and cortisone, which together represent 32.1% of the total immunoreactivity.

In the black bear (Fig. 3F), however, no distinct peak of immunoreactivity could be assigned to cortisone and cortisol, respectively, neither when testing the 21-HS assay nor the 3-CMO assay. This might be due to very low cortisol concentrations in hairs of black bears, as determined by LC-MS/MS (Table 3). The 21-HS assay cross-reacts with unknown compounds that would falsify the results. By contrast, it appeared that the 3-CMO assay detected small amounts of cortisol (the lowest of all species studied so far) which can be suggestively seen at the position of the cortisol standard (Fraction 13) apparently unaffected by other compounds.

### Summary of all HPLC-immunograms: a species comparison

Based on the HPLC immunograms of the investigated species (Fig. 3A–F), the cortisol-3-CMO EIA was determined to be the assay of choice when compared with the corresponding 21-HS assay. An exception might be the black bear where the results were less clear. All results are summarized in Fig. 4, whereby Fractions 11–14 correspond to the sum of cortisone and cortisol, Fractions 3–7 to presumed polar conjugates of GC and Fractions 34–54 to compounds less polar than corticosterone found to be elevated in all HPLC immunograms causing falsification of measurements.

Except in the lynx, both assays detected the proportion of the most polar immunoreactivities in Fractions 3–7 in a similar order. Huge differences existed regarding the unpolar compounds between Fractions 34 and 54, with a distinctly smaller or absent affinity of the 3-CMO assay towards these compounds in all species, simultaneously improving the specificity of the 3-CMO assay towards cortisone and cortisol above 60% in the lynx, the cheetah and the hyena.

## Discussion

Our comparative hGC analysis in six mammalian species demonstrate that results of cortisol EIA may lead to an overestimation of hGCs and that in dependence of chosen immune assay erroneous results might be obtained. The method of choice to exactly quantify hGC is LC-MS/MS (Russell *et al.*, 2015; Carlitz *et al.,* 2016). The LC-MS/MS technique recognizes the analyte by the combined actions of liquid chromatography separation and the specific composition of mass/charge ratio of fragments generated during mass spectrometry allowing the simultaneous quantification of cortisol, cortisone and corticosterone and their differentiation from putative precursors or metabolites and from other steroids, such as testosterone and progesterone. Unlike when using EIA, this high specificity prevents an impact of similar steroids or non-steroidal compounds that may bias the quantification of cortisol, cortisone and corticosterone. In addition, LC-MS/MS methods have shown good reproducibility and high specificity and can be used to analyze multiple steroids simultaneously (Parikh *et al.*, 2018).

Hair has been recognized as a biomaterial that accumulates GC hormones (Davenport *et al.,* 2006; Kirschbaum *et al.*, 2009; Macbeth *et al.,* 2010) and is supposed to reflect average blood serum level. In contrast to faeces, authentic hormones are present within the inert hair matrix as shown for human and many other species, although the precise mechanisms by which steroid hormones are incorporated into hair are still not fully understood (Ito *et al.,* 2005). In addition to systemic origin, a local synthesis of GC by the hair follicle is suggested (Cirillo and Prime, 2011) which may impact the steroid pattern in hairs. Our LC-MS/MS quantification of hGC in different mammalian species revealed different relations between cortisol, cortisone and corticosterone. For the hair of mongoose, hyena and marmots, cortisol is present in identical amounts compared to cortisone, whereas in the Iberian lynx and the cheetah cortisone is almost twice as high as cortisol. Corticosterone was found at very low levels. In the hair of Asiatic black bear, however, corticosterone was identified being the only GC measurable in this species (Table 3). Different serum levels as well as cortisol/corticosterone ratios were also described in different wildlife species (Koren *et al.*, 2012a), which were also unstable across individuals. In addition, diverging hair matrices may also contribute to species specific incorporation and storage patterns (e.g. impact of structural differences, different depositions of oils or pheromones and climatic conditions).

The relation between the different GC in blood serum might be an indicator for endocrine misbalances (Parikh *et al.,* 2018) or physiological stages like hibernation, and the cortisol/cortisone ratio in serum is not necessarily reflected in hGC. In human serum, the cortisol/cortisone ratio is about 5 : 1 (250 ng/ml cortisol vs. 50 ng/ml cortisone) (Eisenhofer *et al.*, 2017), whereas in hairs the relationship was found to be opposite with 7 pg cortisol vs. 25 pg cortisone per mg hair (1 : 3.5) (Stalder *et al.,* 2013). It is known that the cortisol/cortisone ratio is regulated by the enzymes 11*β*-HSD1 and 11*β*-HSD2. While 11*β*-HSD2 is responsible for producing cortisone from cortisol, 11*β*-HSD1 works predominantly in the opposite direction, reducing cortisone to cortisol in the liver, adipose tissue and other tissues (Liu *et al.*, 2019). Thus, the cortisol/cortisone ratio is not determined by GC production from the adrenal, but rather by the metabolic activity of target cells, including hair follicles.

Both EIA presented in this study were in-house made based on a polyclonal antiserum against cortisol, and both assays were also validated for non-invasive monitoring of fGMs. Those against cortisol-3-CMO have been previously utilized to non-invasively monitor changes in adrenal activity in faecal samples from hyenas (Benhaiem *et al.*, 2012); those against cortisol-21-HS were previously used for cortisol analyses in blood (Voigt *et al.*, 2004) and to non-invasively monitor adrenal activity in faeces from bats (Kelm *et al.,* 2016). Antibodies against cortisol-3-CMO are used quite commonly to monitor adrenal activity in several primate species such as Barbary macaques (*Macaca sylvanus*), lowland gorillas (*Gorilla gorilla*) and common marmosets (*Callithrix jacchus*) (Heistermann *et al.*, 2006), and in domestic dogs (*Canis lupus familiaris*) (Schatz *et al.*, 2001). Remarkably, both EIA were found unsuitable to monitor adrenal activity in the Iberian lynx based on fGM (Pribbenow *et al.*, 2014).

Faecal samples from carnivores usually contain only minor amounts of the original hormone (Young *et al.,* 2004); thus, it is not surprising that EIA directed towards the original steroids are unsuitable in some species. In hair extracts, however, we expect to find the non-metabolized hormones, thus cortisol assays should be able to detect cortisol accordingly. Our present data, however, demonstrate that cortisol-EIA based on different antibodies may lead to substantially deviating data, not only in respect of the hormone quantities determined but also providing misleading results. We analyzed the baseline variation in hair cortisol of Egyptian mongooses and highlight the importance of accounting for influences of age, sex and storage time by applying the 3-CMO EIA (Azevedo *et al.,* 2019). Determination of hGC with the 21-HS EIA was performed on the same sample set. When comparing both EIA (Table 1), higher putative amounts of GC were detected when using the cortisol-21-HS test. These results might lead to the assumption that cortisol-21-HS was superior compared to cortisol-3-CMO, but our HPLC immunograms revealed exactly the opposite. A pronounced cross-reactivity towards unknown substances extracted from hair was found to be very obvious, instead of a higher specificity toward hGC (Fig. 3). The results of both assays were significantly correlated (Fig. 1), but when we replicated the models used to the assess the effect of age, sex, season and sample storage time (Azevedo *et al*. 2019), the model using the 21-HS EIA data set did not show an effect of sex and storage time (Table 2, Supplement Table 3).

In the Iberian lynx, we achieved the best concordance between both EIA, with mean levels that are only 2.3 times higher when using the cortisol-21-HS assay (Table 1). Despite this apparently acceptable difference, the HPLC-immunogram showed very similar amounts of cortisol and cortisone (Fig. 3B) but also increasing amounts of unknown compounds between Fractions 33 and 55 with an additional peak in Fraction 34 in case of the cortisol-21-HS assay. Nevertheless, concentrations measured with both EIA do significantly correlate with each other. When applying the cortisol-3-CMO assay, however, the hGC concentrations almost exactly matched with the cortisol + cortisone amount determined by LC-MS/MS, verifying this EIA as more appropriate for hGC analyses in the lynx. In contrast, hair samples collected from Egyptian mongooses and marmots revealed 8- to 10-fold higher hGC concentrations when using the cortisol-21-HS EIA. In addition, comparative HPLC analysis (Fig. 3) confirmed that in these species cortisol-3-CMO was the more appropriate assay, even if hGC concentrations were still double to 3-fold compared to LC-MS/MS cortisol + cortisone amounts (Table 3, Fig. 2), still indicating an overestimation of hGC by this EIA. This agrees with data from Russell *et al*. (2015). They determined hGC in human hairs with four immunoassay methods, which were highly and positively correlated with two LC-MS/MS methods. However, LC-MS/MS hGC concentrations were between 2.5 and 50 times lower in comparison to the EIA results. The authors suggested employing a correction factor for each lab to achieve similar ranges of normal levels.

Our data on hair analysis in wildlife species contribute to the growing data base of studies that aim to use hGC (Koren *et al.*, 2002; Davenport *et al.,* 2006; Accorsi *et al.,* 2008; Comin *et al.*, 2011) to evaluate chronic stress, in most of which assays based on antibodies directed against cortisol have been applied (Koren *et al.*, 2008; Ashley *et al.,* 2011; Carlitz *et al.*, 2014; Peric *et al.*, 2018). According to our results, different antibodies generated different immunograms indicating that besides cortisol and cortisone, unknown immunoreactive compounds can be extracted from hair in different quantities. Additionally, external factors on regions of growing hair may influence the composition of immunoreactive substances (Salaberger *et al.,* 2016). The comparison of HPLC immunograms established from sheep hairs exposed to dexamethasone fluid revealed significantly different compositions, when applying a cortisol-3-CMO and a cortisol-21-HS antibody, respectively.

It might be suggested that the method of steroid extraction influences the outcome of HPLC immunograms. Although we did not compare the two extraction methods for hair cortisol directly, our results indicate that irrespectively of the extraction method, high amounts of interfering unpolar components were determined with the cortisol-21-HS EIA. This applies to hairs from the mongoose and the lynx where hairs were milled and extracted for 30 min as well as from the other four species, where overnight extractions at 45°C were carried out. Independently of the extraction method, all HPLC immunograms were characterized by distinct amounts of interfering unpolar components between Fractions 34–54 (Fig. 3) when using the cortisol-HS-21 EIA, but obviously better results were achieved with the cortisol-3-CMO assay. Best results with more than 60% of immune reactivity contributing to cortisol + cortisone were obtained for hyena and the cheetah (no milling) and for the pooled milled Iberian lynx samples (Fig. 3).

The large amounts of unknown immunoreactivities between Fractions 34 and 54 might be caused by pooling and concentrating the extracts prior to HPLC-analyses, which uses extracts arisen from 500–1000 mg of hairs. Pooling is suggested to enhance inclusion of potential interfering compounds from the matrix (Koren *et al*., 2012b). Against this suggestion argues the parallelism of hair extracts in case of both EIA (data not shown), and the observation that all HPLC-immunograms showed comparable amounts of cortisone and cortisol at their corresponding elution positions (Fraction 11 and Fraction 13/14, respectively) when applying both cortisol EIA (Fig. 3). The unknown unpolar compounds, however, were exclusively detected by the cortisol-21-HS EIA. These unknown immunoreactivities lead to a substantial overestimation of hGC concentration when using the cortisol-21-HS EIA, which was up to 12-fold for African black bear (Table 1). Similar results were obtained by Kroshko *et al*. (2017) when quantifying hGC in grizzly bears applying different commercial ELISA kits commonly used for hGC determinations. Mean concentrations between 0.70 and 9.35 pg/mg of hairs were measured, resulting in a difference up to 13-fold between kits. Because nearly all commercial ELISA kits are originally designed for diagnostic use in plasma or serum, adapting them for quantification of hGC introduces potential for cross-reactions of unknown substances extracted from hair as shown in our comparison between the HPLC immunograms. Thus, absolute hGC values amongst studies of the same species that use different ELISAs should be viewed with caution.

As a result of our study, we decided to use the cortisol-3-CMO EIA for hGC analysis of mammalian hair extracts. In four of five tested species (mongoose, Iberian lynx, hyena, marmot), the determined amount of hGC nicely correlated with the LC-MS/MS determination of cortisol + cortisone, despite the overestimations in the mongoose, the hyena and the marmot (Table 3). This was not the case in the black bear where both EIA failed to produce reasonable results, possibly due to a very low content of hGC. The suitability of the 3-CMO EIA was demonstrated based on its strong specificity towards cortisone and cortisol, and low cross-reaction with other (unknown) mainly unpolar compounds extracted from hair. Distinct species differences not only in mean hGC concentration but also in the cortisol/cortisone ratio were demonstrated by both LC-MS/MS and HPLC immunograms.

Although we just used two different cortisol EIA, overall recommendation towards EIA selection for hGC analysis in wildlife can be deducted. In all investigated species, except the Asiatic black bear, we show that cortisone and cortisol were simultaneously present in hair extracts. Consequently, an appropriate EIA should cross-react to at least these two GC to account for any change of the cortisol/cortisone ratio in respect to physiological changes (Koren *et al.*, 2012a, Parikh *et al.,* 2018) or species-specific mechanisms of GC incorporation and storage within hair matrices. A selected antibody (EIA) should also express negligible affinity towards unknown substances that are less polar than corticosterone probably generating overestimations that could be misinterpreted as increased adrenocortical activity. Exemplarily for the mongoose we demonstrated that choosing the wrong EIA for hGC analyses lead to results that confound interpretation of adrenocortical activity. This validation can only performed by applying HPLC immunograms. Finally and most importantly, for any non-invasive stress related analysis, a proper pharmacological or biological validation is inevitable to demonstrate that data obtained are in relation to the adrenocortical activity (stress). Irrespective the source of hair GC, local or systemic, experiments in dairy cattle (Gonzalez-de-la-Vara *et al.,* 2011), goats (Endo *et al.*, 2018), Canada lynx (Terwissen *et al.,* 2013) and chipmunks (Mastromonaco *et al.,* 2014) showed that repeated ACTH treatments from 2 weeks to 2 months were sufficient to affect hGC. However, HPA activation by a single ACTH doses has been shown to be insufficient (Ashley *et al.,* 2011). This clearly supports hGC as a biomarker of repeated or even long-term stress events. The use of validated EIA or even the gold standard LC-MS/MS will generate more studies on wild animals and will also elucidate further species specific differences for this attractive non-invasive approach.

## Supplementary Material

Suppl_table_1_coaa009

Suppl_table_2_coaa009

## References

[ref1] Accorsi PA, Carloni E, Valsecchi P, Viggiani R, Gamberoni M, Tamanini C, Seren E (2008) Cortisol determination in hair and feces from domestic cats and dogs. Gen Comp Endocrinol 155: 398–402. 17727851 10.1016/j.ygcen.2007.07.002

[ref2] Acker M, Mastromonaco G, Schulte-Hostedde AI (2018) The effects of body region, season and external arsenic application on hair cortisol concentration. Conserv Physiol 6: coy037. doi: 10.1093/conphys/coy037 PMC6041973 30018762

[ref3] Ashley NT, Barboza PS, Macbeth BJ, Janz DM, Cattet MR, Booth RK, Wasser SK (2011) Glucocorticosteroid concentrations in feces and hair of captive caribou and reindeer following adrenocortico tropic hormone challenge. Gen Comp Endocrinol 172: 382–391. 21501613 10.1016/j.ygcen.2011.03.029

[ref4] Azevedo A, Bandeira V, Dehnhard M, Bailey L, Fonseca C, de Sousa L, Jewgenow K (2019) Age, sex and storage time influence hair cortisol levels in a wild mammal population**,** PLoS One 14:e0222963. doi: 10.1371/journal.pone.0222963 PMC6688795 31398221

[ref5] Bandeira V, Virgós E, Barros T, Cunha MV, Fonseca C (2016) Geographic variation and sexual dimorphism in body size of the Egyptian mongoose, *Herpestes ichneumon* in the western limit of its European distribution. J Comp Zool 264: 1–10.

[ref6] Bauer CM, Hayes LD, Ebensperger LA, Romero LM (2014) Seasonal variation in the degu (Octodon degus) endocrine stress response. Gen Comp Endocrinol 197: 26–32. 24321176 10.1016/j.ygcen.2013.11.025

[ref7] Bates D, Mächler M, Bolker B, Walker S (2015) Fitting linear mixed-effects models using lme4. J Stat Softw [Internet].

[ref8] Benhaiem S, Dehnhard M, Bonanni R, Hofer H, Goymann W, Eulenberger K, East ML (2012) Validation of an enzyme immunoassay for the measurement of fecal glucocorticoid metabolites in spotted hyenas (Crocuta crocuta). Gen Comp Endocrinol 178: 265–271. 22634955 10.1016/j.ygcen.2012.05.006

[ref9] Carlitz EH, Kirschbaum C, Stalder T, Schaik CP van (2014) Hair as a long-term retrospective cortisol calendar in orang-utans (Pongo spp.): new perspectives for stress monitoring in captive management and conservation. Gen Comp Endocrinol 195: 151–156. 24239791 10.1016/j.ygcen.2013.11.002

[ref10] Carlitz EH, Miller R, Kirschbaum C, Gao W, Hanni DC, van Schaik CP (2016) Measuring hair cortisol concentrations to assess the effect of anthropogenic impacts on wild chimpanzees (Pan troglodytes). PLoS One 11: e0151870. 27050418 10.1371/journal.pone.0151870 PMC4822880

[ref11] Carnes M, Kalin NH, Lent SJ, Barksdale CM, Brownfield MS (1988) Pulsatile acth secretion: variation with time of day and relationship to cortisol. Peptides 9: 325–331. 2836825 10.1016/0196-9781(88)90268-9

[ref12] Cirillo N, Prime SS (2011) Keratinocytes synthesize and activate cortisol. J Cell Biochem 112: 1499–1505. 21344493 10.1002/jcb.23081

[ref13] Comin A, Prandi A, Peric T, Corazzin M, Dovier S, Bovolenta S (2011) Hair cortisol levels in dairy cows from winter housing to summer highland grazing. Livest Sci 138: 69–73.

[ref15] Davenport MD, Tiefenbacher S, Lutz CK, Novak MA, Meyer JS (2006) Analysis of endogenous cortisol concentrations in the hair of rhesus macaques. Gen Comp Endocrinol 147: 255–261. 16483573 10.1016/j.ygcen.2006.01.005

[ref16] Dehnhard M, Fanson K, Frank A, Naidenko SV, Vargas A, Jewgenow K (2010) Comparative metabolism of gestagens and estrogens in the four lynx species, the eurasian (Lynx lynx), the iberian (L. Pardinus), the Canada lynx (L. Canadensis) and the bobcat (L. Rufus). Gen Comp Endocrinol 167: 287–296. 20346945 10.1016/j.ygcen.2010.03.023

[ref17] Eisenhofer G et al. (2017) Reference intervals for plasma concentrations of adrenal steroids measured by LC-MS/MS: impact of gender, age, oral contraceptives, body mass index and blood pressure status. Clin Chim Acta 470: 115–124. 28479316 10.1016/j.cca.2017.05.002 PMC5504266

[ref18] Endo N, Yamane H, Rahayu LP, Tanaka T (2018) Effect of repeated adrenocorticotropic hormone administration on reproductive function and hair cortisol concentration during the estrous cycle in goats. Gen Comp Endocrinol 259: 207–212. 29199102 10.1016/j.ygcen.2017.11.027

[ref19] Finkenwirth C, Jewgenow K, Meyer HH, Vargas A, Dehnhard M (2010) Pgfm (13,14-dihydro-15-keto-pgf(2alpha)) in pregnant and pseudo-pregnant Iberian lynx: a new noninvasive pregnancy marker for felid species. Theriogenology 73: 530–540. 20022361 10.1016/j.theriogenology.2009.10.008

[ref20] Gao W, Stalder T, Foley P, Rauh M, Deng H, Kirschbaumb C (2013) Quantitative analysis of steroid hormones in human hair using a column-switching LC–APCI–MS/MS assay. J Chromatogr B 928: 1–8. 10.1016/j.jchromb.2013.03.008 23584040

[ref21] Gonzalez-de-la-Vara MR, Valdez RA, Lemus-Ramirez V, Vazquez-Chagoyan JC, Villa-Godoy A, Romano MC (2011) Effects of adrenocorticotropic hormone challenge and age on hair cortisol concentrations in dairy cattle. Can J Vet Res 75: 216–221. 22210998 PMC3122973

[ref22] Halekoh U, Højsgaard S (2014) A Kenward-Roger approximation and parametric bootstrap methods for tests in linear mixed models the R package pbkrtest. J Stat Softw [Internet].

[ref23] Heistermann M, Palme R, Ganswindt A (2006) Comparison of different enzyme-immunoassays for assessment of adrenocortical activity in primates based on fecal analysis. Am J Primatol 68: 257–273. 16477600 10.1002/ajp.20222

[ref24] Ito N, Ito T, Kromminga A, Bettermann A, Takigawa M, Kees F, Straub RH, Paus R (2005) Human hair follicles display a functional equivalent of the hypothalamic-pituitary-adrenal axis and synthesize cortisol. FASEB J 19: 1332–1334. 15946990 10.1096/fj.04-1968fje

[ref25] Kapoor A, Schutz-Darken N, Ziegler TE (2018) Radiolabel validation of cortisol in the hair of rhesus monkeys. Psychoneuroendocrinology 97: 190–195. 30053699 10.1016/j.psyneuen.2018.07.022 PMC6138524

[ref26] Keckeis K, Lepschy M, Schopper H, Moser L, Troxler J, Palme R (2012) Hair cortisol: a parameter of chronic stress? Insights from a radiometabolism study in guinea pigs. J Comp Physiol B 182: 985–996. 22592890 10.1007/s00360-012-0674-7

[ref27] Kelm DH, Popa-Lisseanu AG, Dehnhard M, Ibanez C (2016) Non-invasive monitoring of stress hormones in the bat Eptesicus isabellinus - do fecal glucocorticoid metabolite concentrations correlate with survival? Gen Comp Endocrinol 226: 27–35. 26673871 10.1016/j.ygcen.2015.12.003

[ref28] Kirschbaum C, Tietze A, Skoluda N, Dettenborn L (2009) Hair as a retrospective calendar of cortisol production-increased cortisol incorporation into hair in the third trimester of pregnancy. Psychoneuroendocrinology 34: 32–37. 18947933 10.1016/j.psyneuen.2008.08.024

[ref29] Koren L, Mokady O, Geffen E (2008) Social status and cortisol levels in singing rock hyraxes. Horm Behav 54: 212–216. 18423638 10.1016/j.yhbeh.2008.02.020

[ref30] Koren L, Mokady O, Karaskov T, Klein J, Koren G, Geffen E (2002) A novel method using hair for determining hormonal levels in wildlife. Anim Behav 63: 403–406.

[ref31] Koren L, Whiteside D, Fahlmann S, Ruckstuhl K, Kutz S, Checkley S, Dumond M, Wynne-Edwards K (2012a) Cortisol and corticosterone independence in cortisol-dominant wildlife. Gen Comp Endocrinol 177: 113–119. 22449618 10.1016/j.ygcen.2012.02.020

[ref32] Koren L, Ng ES, Soma KK, Wynne-Edwards KE (2012b) Sample preparation and liquid chromatography-tandem mass spectrometry for multiple steroids in mammalian and avian circulation. PLoS One 8 1: e32496. 10.1371/journal.pone.0032496 PMC3288106 22384262

[ref33] Kroshko T, Kapronczai L, Cattet MR, Macbeth BJ, Stenhouse GB, Obbard ME, Janz DM (2017) Comparison of methanol and isopropanol as wash solvents for determination of hair cortisol concentration in grizzly bears and polar bears. MethodsX 4: 68–75. 28203534 10.1016/j.mex.2017.01.004 PMC5295503

[ref34] Liu X et al. (2019) Gender-specific independent and combined effects of the cortisol-to-cortisone ratio and 11-deoxycortisol on prediabetes and type 2 diabetes mellitus: from the Henan rural cohort study. J Diabetes Res 2019: 4693817. 31281850 10.1155/2019/4693817 PMC6589245

[ref35] Ludwig C, Dehnhard M, Pribbenow S, Silinski-Mehr S, Hofer H, Wachter B (2019) Asymmetric reproductive aging in cheetah (*Acinonyx jubatus*) females in European zoos. J Zoo Aquarium Res 7: 87–93.

[ref36] Ludwig C, Wachter B, Silinski-Mehr S, Ganswindt A, Bertschinger H, Hofer H, Dehnhard M (2013) Characterisation and validation of an enzyme-immunoassay for the non-invasive assessment of fecal glucocorticoid metabolites in cheetahs (Acinonyx jubatus). Gen Comp Endocrinol 180: 15–23. 23108105 10.1016/j.ygcen.2012.10.005

[ref37] Macbeth HBJ, Cattet MRL, Stenhouse GB, Gibeau ML, Janz DM (2010) Hair cortisol concentration as a noninvasive measure of long-term stress in free-ranging grizzly bears (ursus arctos): considerations with implications for other wildlife. Can J Zool 88: 935–949.

[ref38] Mastromonaco GF, Gunn K, McCurdy-Adams H, Edwards DB, Schulte-Hostedde AI (2014) Validation and use of hair cortisol as a measure of chronic stress in eastern chipmunks (tamias striatus). Conserv Physiol 2: cou055. 27293676 10.1093/conphys/cou055 PMC4732495

[ref39] McEwen BS, Wingfield JC (2003) The concept of allostasis in biology and biomedicine. Horm Behav 43: 2–15. 12614627 10.1016/s0018-506x(02)00024-7

[ref40] Mengulluoglu D, Fickel J, Hofer H, Forster DW (2019) Non-invasive fecal sampling reveals spatial organization and improves measures of genetic diversity for the conservation assessment of territorial species: Caucasian lynx as a case species. PLoS One 14: e0216549. 31075125 10.1371/journal.pone.0216549 PMC6510455

[ref41] Mohd Salleh F et al. (2017) An expanded mammal mitogenome dataset from southeast asia. Gigascience 6: 1–8. 10.1093/gigascience/gix053 PMC5737531 28873965

[ref42] Palme R (2019) Non-invasive measurement of glucocorticoids: advances and problems. Physiol Behav 199: 229–243. 30468744 10.1016/j.physbeh.2018.11.021

[ref43] Parikh TP, Stolze BR, Ozarda Ilcol Y, Jonklaas J, Welsh K, Masika LS, Hill MJ, DeCherney AH, Soldin SJ (2018) Diurnal variation of steroid hormones and reference intervals using mass spectrometric analysis. Endocr Connect. 10.1530/EC-18-0417 PMC6280590 30400040

[ref45] Peric T, Comin A, Corazzin M, Montillo M, Canavese F, Stebel M, Prandi A (2018) Hair cortisol concentrations in New Zealand white rabbits subjected to surgery. Animal Welfare 27: 13–20. 10.1080/10888705.2016.1183489 27191037

[ref47] Pribbenow S, East ML, Ganswindt A, Tordiffe AS, Hofer H, Dehnhard M (2015) Measuring fecal epi-androsterone as an indicator of gonadal activity in spotted hyenas (crocuta crocuta). PLoS One 10: e0128706. 26107516 10.1371/journal.pone.0128706 PMC4481319

[ref48] Pribbenow S, Jewgenow K, Vargas A, Serra R, Naidenko S, Dehnhard M (2014) Validation of an enzyme immunoassay for the measurement of fecal glucocorticoid metabolites in eurasian (lynx lynx) and iberian lynx (lynx pardinus). Gen Comp Endocrinol 206: 166–177. 25066418 10.1016/j.ygcen.2014.07.015

[ref49] Raul JS, Cirimele V, Ludes B, Kintz P (2004) Detection of physiological concentrations of cortisol and cortisone in human hair. Clin Biochem 37: 1105–1111. 15589817 10.1016/j.clinbiochem.2004.02.010

[ref50] Romero LM (2002) Seasonal changes in plasma glucocorticoid concentrations in free-living vertebrates. Gen Comp Endocrinol 128: 1–24. 12270784 10.1016/s0016-6480(02)00064-3

[ref51] Russell E, Kirschbaum C, Laudenslager ML, Stalder T, de Rijke Y, van Rossum EF, Van Uum S, Koren G (2015) Toward standardization of hair cortisol measurement: results of the first international interlaboratory round robin. Ther Drug Monit 37: 71–75. 25387254 10.1097/FTD.0000000000000148

[ref52] Salaberger T, Millard M, Makarem SE, Mostl E, Grunberger V, Krametter-Frotscher R, Wittek T, Palme R (2016) Influence of external factors on hair cortisol concentrations. Gen Comp Endocrinol 233: 73–78. 27167500 10.1016/j.ygcen.2016.05.005

[ref53] Schatz S, Palme R (2001) Measurement of fecal cortisol metabolites in cats and dogs: a non-invasive method for evaluating adrenocortical function. Vet Res Commun 25: 271–287. 11432429 10.1023/a:1010626608498

[ref54] Sheriff MJ, Dantzer B, Delehanty B, Palme R, Boonstra R (2011) Measuring stress in wildlife: techniques for quantifying glucocorticoids. Oecologia 166: 869–887. 21344254 10.1007/s00442-011-1943-y

[ref55] Stalder T, Kirschbaum C, Alexander N, Bornstein SR, Gao W, Miller R, Stark S, Bosch JA, Fischer JE (2013) Cortisol in hair and the metabolic syndrome. J Clin Endocrinol Metab 98: 2573–2580. 23585660 10.1210/jc.2013-1056

[ref56] Terio KA, Citino SB, Brown JL (1999) Fecal cortisol metabolite analysis for noninvasive monitoring of adrenocortical function in the cheetah (acinonyx jubatus). J Zoo Wildl Med 30: 484–491. 10749432

[ref57] Terwissen CV, Mastromonaco GF, Murray DL (2013) Influence of adrenocorticotrophin hormone challenge and external factors (age, sex, and body region) on hair cortisol concentration in Canada lynx (lynx canadensis). Gen Comp Endocrinol 194: 162–167. 24080086 10.1016/j.ygcen.2013.09.010

[ref58] Tilbrook AJ, Canny BJ, Serapiglia MD, Ambrose TJ, Clarke IJ (1999) Suppression of the secretion of luteinizing hormone due to isolation/restraint stress in gonadectomised rams and ewes is influenced by sex steroids. J Endocrinol 160: 469–481. 10076193 10.1677/joe.0.1600469

[ref59] Touma C, Palme R, Sachser N (2004) Analyzing corticosterone metabolites in fecal samples of mice: a noninvasive technique to monitor stress hormones. Horm Behav 45: 10–22. 14733887 10.1016/j.yhbeh.2003.07.002

[ref60] Voigt CC, Fassbender M, Dehnhard M, Wibbelt G, Jewgenow K, Hofer H, Schaub GA (2004) Validation of a minimally invasive blood-sampling technique for the analysis of hormones in domestic rabbits, oryctolagus cuniculus (lagomorpha). Gen Comp Endocrinol 135: 100–107. 14644649 10.1016/j.ygcen.2003.08.005

[ref61] Wingfield JC, Sapolsky RM (2003) Reproduction and resistance to stress: when and how. J Neuroendocrinol 15: 711–724. 12834431 10.1046/j.1365-2826.2003.01033.x

[ref62] Young KM, Walker SL, Lanthier C, Waddell WT, Monfort SL, Brown JL (2004) Noninvasive monitoring of adrenocortical activity in carnivores by fecal glucocorticoid analyses. Gen Comp Endocrinol 137: 148–165. 15158127 10.1016/j.ygcen.2004.02.016

